# Optimizing healthcare resources in pyogenic liver abscess: a dual-threshold HDL-CRP model for predicting hospitalization duration across multi-cohorts

**DOI:** 10.3389/fmed.2026.1708360

**Published:** 2026-04-30

**Authors:** Mingzhu Tao, Muye Xia, Suling Chen, Guichan Liao, Jingchun Mao, Shaohang Cai, Jie Peng, Xuwen Xu

**Affiliations:** 1 Department of Infectious Diseases, Nanfang Hospital, Southern Medical University, Guangzhou, China; 2 State Key Laboratory of Organ Failure Research, Guangzhou, China; 3 Key Laboratory of Infectious Diseases Research in South China (Southern Medical University), Ministry of Education, Guangzhou, China; 4 Guangdong Provincial Key Laboratory for Prevention and Control of Major Liver Diseases, Guangzhou, China; 5 Guangdong Provincial Clinical Research Center for Viral Hepatitis, Guangzhou, China; 6 Guangdong Institute of Hepatology, Guangzhou, China; 7 Guangdong Provincial Research Center for Liver Fibrosis Engineering and Technology, Guangzhou, China; 8 Department of Infection Disease, Maoming People’s Hospital, Maoming, China; 9 Center of Scientific Research, Maoming People’s Hospital, Maoming, China

**Keywords:** C-reactive protein, HDL cholesterol, hospitalization, prediction model, pyogenic liver abscess, resource allocation, risk stratification

## Abstract

**Background:**

Prolonged hospitalization for pyogenic liver abscess (PLA) burdens patients and health systems. We examined whether admission high-density lipoprotein cholesterol (HDL-C) predicts length of stay (LOS) and whether a simple HDL–C-reactive protein (CRP) dual-threshold can flag patients at risk of prolonged stay.

**Methods:**

We analyzed a prospective adult PLA cohort at a tertiary center (2018–2023; *n* = 138) and validated findings in MIMIC-IV ICU patients with liver abscess (*n* = 38) and in NHANES 2017–2020 (*n* = 9,226). Multivariable models related admission HDL-C to log-transformed LOS; model performance and calibration were assessed with internal resampling and external validation. We further evaluated a dual-threshold rule and conducted mediation analysis with CRP.

**Results:**

Lower HDL-C independently associated with longer LOS. A nomogram combining HDL-C, abscess size, and sepsis performed well (*R*^2^≈0.66; RMSE≈6.4 d) and remained directionally consistent across external datasets. A dual-threshold (HDL-C < 1.03 mmol/L and CRP > 1.0 mg/dL) identified a high-risk subgroup with greater odds of prolonged stay. CRP mediated a small proportion of the HDL-C–LOS association.

**Conclusion:**

Admission HDL-C, particularly when interpreted together with CRP, may help identify PLA patients at increased risk of prolonged hospitalization at the time of admission. Patients with low HDL-C and high CRP may warrant closer monitoring, earlier evaluation for source control or drainage, and more proactive inpatient planning. Prospective implementation studies are warranted.

## Introduction

Pyogenic liver abscess (PLA) is a clinically important intra-abdominal infection (1) associated with substantial morbidity, prolonged hospitalization, and non-negligible mortality despite advances in imaging-guided drainage and antimicrobial therapy (2–4). Common predisposing factors include biliary tract disease, diabetes mellitus, older age, and immunocompromised status (2, 4, 5). Clinical course is highly heterogeneous, ranging from localized infection to sepsis and septic shock, and adverse outcomes are more likely in patients with advanced age, major comorbidities, marked physiological derangement, and severe abscess-related complications (5–7). This heterogeneity creates a clear need for simple admission-time tools that can identify patients at increased risk of prolonged hospitalization and more complicated clinical courses (5, 7).

Common causative organisms of PLA include Klebsiella pneumoniae and other enteric Gram-negative pathogens (8–10), while the emergence of multidrug-resistant organisms further complicates empirical antimicrobial selection and clinical management (9, 11). Previous prognostic studies in PLA have mainly emphasized anatomical burden, procedural factors (12), microbiological characteristics (6, 7, 13, 14), or severe outcomes such as sepsis, metastatic infection, or mortality. However, these approaches are less suited to simple admission-time prediction of hospital length of stay and generally do not incorporate host-response biomarkers such as HDL-C (15–17). Accordingly, clinically practical tools that link admission-time biological information to hospitalization burden remain limited.

Among candidate admission biomarkers, HDL-C was of particular interest because it was obtained as part of the routine admission laboratory panel rather than selected as a specialized exploratory marker. This made HDL-C a clinically practical candidate for early risk stratification (17, 18) at the time of admission, as it is inexpensive, readily available, and easy to interpret. In addition, HDL-C has biologically plausible links to host inflammatory and immune responses (16–19), while its value for predicting hospitalization burden in PLA has not been adequately evaluated. We therefore aimed to determine whether admission HDL-C predicts LOS in PLA, to develop a simple triage rule incorporating CRP, and to evaluate the generalizability of these findings across external datasets.

## Study participants and methods

### Study design and inclusion criteria

The reporting of this study was informed by the TRIPOD+AI statement (20). This study integrated multi-source data, comprising a prospective cohort, a retrospective validation cohort, and a population-level cohort, to investigate the role of HDL in modulating systemic inflammation and clinical outcomes in patients with liver abscess. The prospective cohort included 138 consecutive adult patients admitted between January 2018 and December 2023 to the Department of Infectious Diseases at Nanfang Hospital, Southern Medical University, a tertiary center in Guangzhou, Guangdong, China. Inclusion criteria required participants to be adults (≥18 years) with pyogenic liver abscess (PLA) diagnosed on the basis of a compatible clinical presentation (2, 21), supportive laboratory evidence of infection or inflammation, and radiologic evidence of hepatic abscess on ultrasound or CT. Microbiological results from blood culture and/or abscess aspirate culture were incorporated when available to support the diagnosis and etiologic classification. Participants were also required to have complete records of admission laboratory testing and hospitalization outcomes. For the prospective cohort, laboratory biomarkers were defined as the first measurements obtained within 24 h of hospital admission as part of the routine admission laboratory panel, including lipid profile (with HDL-C), inflammatory markers (such as CRP and IL-6), routine hematologic indices, and liver and renal function tests. In the prospective cohort, glucose was defined as the first blood glucose measurement obtained after hospital admission, and diabetes referred to a pre-existing documented history of diabetes mellitus. Exclusion criteria included malignancy, autoimmune disease, end-stage organ failure, non-bacterial liver abscess (e.g., amoebic abscess or cystic lesions), and missing critical data (>10% of variables). Ethical approval was obtained from the Ethics Committee of Nanfang Hospital, Southern Medical University (NFEC-2021-355), and written informed consent was obtained from all participants. Microbiological data were obtained from routine reports issued by the hospital microbiology laboratory. Blood cultures were collected at admission when clinically indicated, and abscess aspirate or drainage fluid cultures were reviewed for patients who underwent percutaneous aspiration or drainage. Organism identification and antimicrobial susceptibility testing were performed by the hospital laboratory according to routine institutional procedures in use during the study period. These microbiological results were recorded to support etiologic classification when available. The retrospective validation cohort utilized data from the MIMIC-IV database (v2.2) (22), a publicly available database of critically ill adult patients in the United States. Liver abscess cases were identified using ICD-10 (K75.0) and ICD-9 (572.0) codes through SQL queries executed in Navicat Premium v16.2. Patients with extreme hospitalization durations ( < 2 d or > 70 d) or missing key variables (HDL, CRP, LOS) were excluded, resulting in 38 eligible cases (Supplementary Figure 4). Variables extracted included hospital length of stay (LOS), the first measured HDL-C level within 24 h of ICU admission, CRP, glucose, diabetes status, and sepsis diagnosis. In the retrospective validation cohort, glucose was defined as the first blood glucose measurement obtained after ICU admission, and diabetes referred to a documented pre-existing diagnosis of diabetes mellitus when available For the retrospective validation cohort, HDL-C was defined as the first available value within 24 h of ICU admission. Baseline demographic characteristics of the included MIMIC-IV cohort are summarized in Supplementary Table 1. In our institution, patients admitted with suspected liver abscess undergo a standardized laboratory panel that includes complete blood count, liver and renal function tests, lipid profile (including HDL-C), and inflammatory markers (including CRP) as part of routine clinical care. Therefore, exclusion due to missing HDL-C or CRP data was minimal (<5%) and is unlikely to have introduced substantial selection bias. The population-level cohort leveraged NHANES 2017–2020 data (23). Hospital length of stay (LOS) was defined using the variable “HUQ071” (days in hospital) from the Hospital Utilization and Access to Care questionnaire. For participants with multiple hospitalizations, the longest stay within the recall period was used. Participants were restricted to adults aged ≥ 18 years. Those with missing LOS data or extreme LOS (>90 d) were excluded (Supplementary Figure 5). This cohort was used to explore whether the inverse relationship between HDL and hospitalization duration could be generalized beyond a specific infectious context.

### Data management and software tools

Data extraction and preprocessing were tailored to each cohort. For the MIMIC-IV database, Navicat Premium v16.2 facilitated SQL query execution and structured data export. Our external validation in the MIMIC-IV cohort, though statistically significant, was limited by a small sample size (n = 38), which affects the precision of the estimates. Future validation in larger, multi-center critical care cohorts is warranted The prospective cohort data were extracted from electronic health records and formatted into standardized tables, while NHANES data were sourced from publicly available datasets. Statistical analyses were conducted using R (version 4.3.0). Data cleaning employed the tidyverse package, with missing values (<5% in the prospective cohort) addressed via multiple imputations (mice package). Continuous variables such as LOS were log-transformed to meet normality assumptions. machine learning models, including Random Forest (randomForest package) and LASSO regression (glmnet package), were implemented to identify key predictors. Mediation analysis utilized the mediation package with 1,000 bootstrapped samples, and visualizations were generated using ggplot2.

#### Machine learning implementation details

1. Random forest specification:

Number of trees: 500 (ntree = 500)

Variables per split: 3 (mtry = floor(sqrt(12)))

Importance metric: % Increase in MSE (%IncMSE)

2. LASSO regression:

glmnet R package v4.1–8

λ selection: 5-fold cross-validation minimizing MSE

Optimal λ: 0.05 (lambda.min)

3. Internal validation:

1000 bootstrap resamples for optimism correction

4. Missing data handling:

Prospective cohort missing rate: HDL 2.1% (3/138), CRP 3.6% (5/138)

Imputed using mice R package (predictive mean matching, 5 imputations)

### Outcome measures and statistical analysis

The primary outcome was hospital length of stay (LOS). LOS was analyzed as a continuous variable after log-transformation. Sepsis referred to sepsis present at admission and was defined using Sepsis-3 criteria (24) in the prospective cohort and ICD-based coding in the MIMIC-IV cohort. Multivariable linear regression was used to assess the association between HDL and LOS, adjusting for age, diabetes status, abscess size (prospective cohort), and other relevant covariates. External validation involved testing HDL–LOS models from the prospective cohort in MIMIC-IV and NHANES. Sensitivity analyses excluded extreme LOS cases (>70 days in MIMIC-IV) and confounding conditions (e.g., HIV in the prospective cohort).

HDL cut-off (1.03 mmol/L) was defined by maximizing Youden’s index in ROC analysis for hospitalization duration > 14 days . The optimal dual-threshold for HDL (< 1.03 mmol/L) and CRP (> 1.0 mg/dL) was determined by maximizing Youden’s index (J) in a receiver operating characteristic (ROC) analysis for predicting prolonged hospitalization (>14 days) in the primary cohort

### Microbiological specimen collection and processing

Blood and pus specimens were collected for culture when clinically indicated. Blood samples were inoculated into aerobic and anaerobic bottles and incubated in the BACT/ALERT^®^ 3D automated blood culture system (BioMérieux, Marcy-l’Étoile, France) until signaled positive or for a maximum of 5 days. Bacterial identification and antibiotic susceptibility testing were conducted in accordance with the guidelines of the Clinical and Laboratory Standards Institute (CLSI). Microorganisms were identified via Matrix-Assisted Laser Desorption/Ionization-Time of Flight Mass Spectrometry (MALDI-TOF MS, BioMérieux, Marcy-l’Etoile, France). Antibiotic susceptibility was assessed using the BD Phoenix™ M50 ID/AST system (Becton Dickinson, Franklin Lakes, NJ) and confirmed by the Kirby-Bauer disk diffusion method on Müller-Hinton agar (Oxoid, Basingstoke, United Kingdom), with results reinterpreted in accordance with CLSI standards (2025). Quality control was ensured using reference strains including *Staphylococcus aureus* ATCC 25923, *Escherichia coli* ATCC 25922, and Pseudomonas aeruginosa ATCC 27853. Antimicrobial susceptibility of rare organisms and specific antimicrobial agents was determined by E-test (bioMérieux, France) according to the manufacturer’s instructions.

### Strengths of the study design

The study’s rigorous design ensured internal validity through precise case definitions (excluding non-bacterial abscesses) and robust data harmonization across cohorts. The integration of prospective clinical data, ICU validation (MIMIC-IV), and population-level insights (NHANES) provided a comprehensive understanding of HDL’s role in inflammation and hospitalization outcomes. Advanced tools like Navicat and R enhanced reproducibility, while a focus on clinically actionable thresholds (e.g., HDL-C < 1.03 mmol/L) underscored translational relevance for personalized care strategies.

### Ethical approval

The study protocol was approved by the NANFANG HOSPITAL SOUTHERN MEDICAL UNIVERSITY, with informed consent for retrospective analysis of de-identified data. NHANES & MIMIC IV was approved by the CDC/NCHS Ethics Review Board and all individuals signed informed consent to participate in NHANES IV

## Results

### Primary cohort findings: baseline and correlations

**FIGURE 1:**
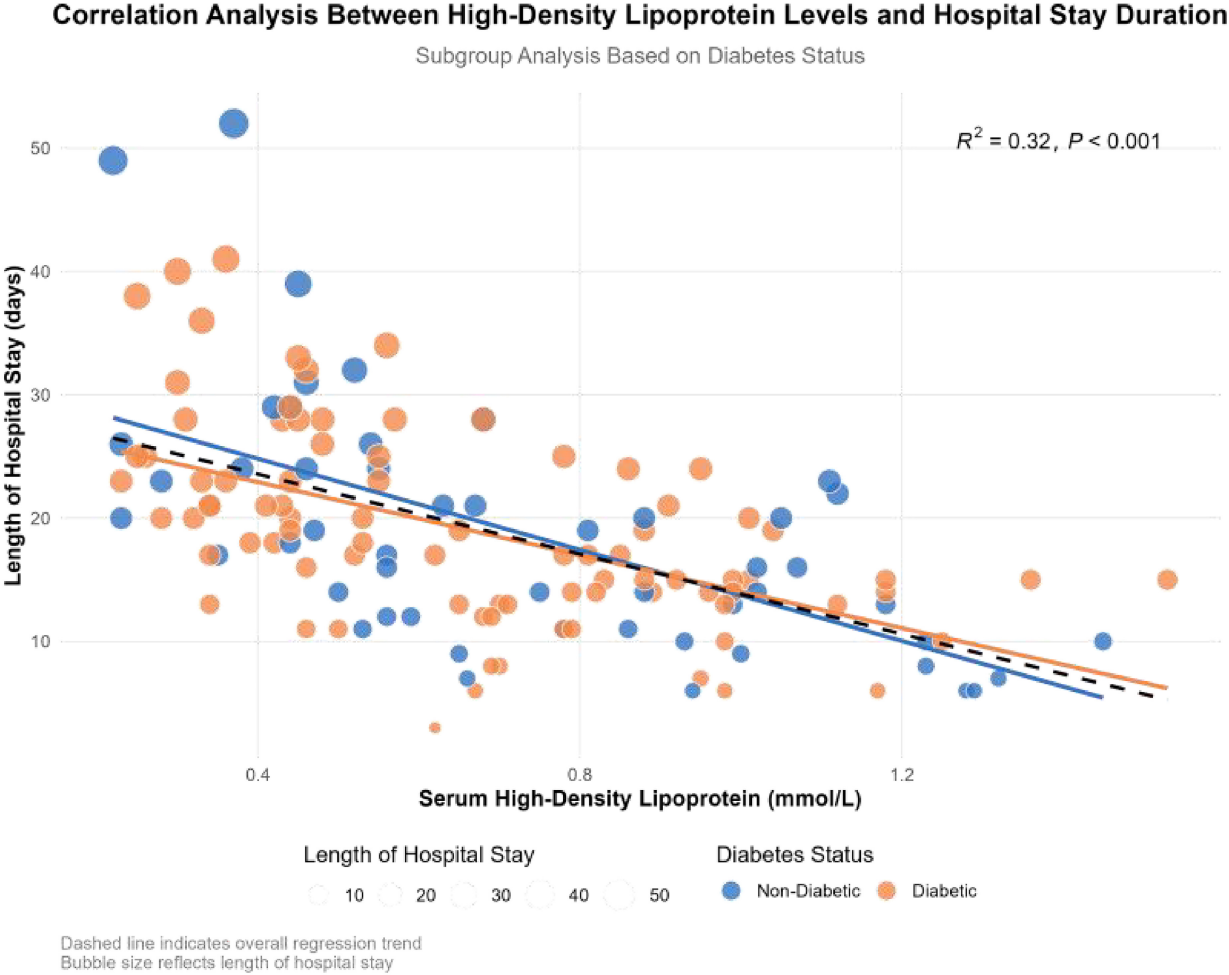
Association between HDL and hospital length of stay.

A total of 138 patients with pyogenic liver abscess were included in the primary cohort. The mean age was 55.2 ± 13.1 years, and 62% had diabetes. Baseline characteristics of the cohort are summarized in Table 1. HDL levels were inversely associated with hospital length of stay (LOS) (*R*^2^ = 0.32, *p* < 0.001; Figure 1). Figure 1 also shows the distribution of patients according to diabetes status.

**TABLE 1:** Baseline characteristics of the study population.

Variable	Value (*n* = 138) n (%)/mean ± SD
Characteristic demographics	
Age (years)	55.159 ± 13.14
Female sex	40 (28%)
Underlying condition	
Diabetes	86 (62%)
Gallbladder and biliary tract diseases	49 (35%)
Cholecystolithiasis	24 (17%)
Cholecystitis	37 (26%)
Biliary calculi cholangitis	11 (8%)
Chronic HBV	19 (13%)
HIV	3 (2%)
Gastrointestinal surgery	3 (2%)
Other malignancy	6 (4%)
Intestinal diseases	37 (26%)
Co-morbidities	
Pneumonia with lung abscess	65 (47%)
Pleurisy	11 (8%)
Renal abscess/perinephric infection	23 (17%)
Peritonitis	18 (13%)
Splenic abscess	1 (0.7%)
Retroperitoneal lymph nodes	9 (6.5%)
Muscular abscess	3 (2%)
Osteomyelitis	2 (1%)
Endophthalmitis	4 (3%)
Brain abscess	1 (0.7%)

**FIGURE 2:**
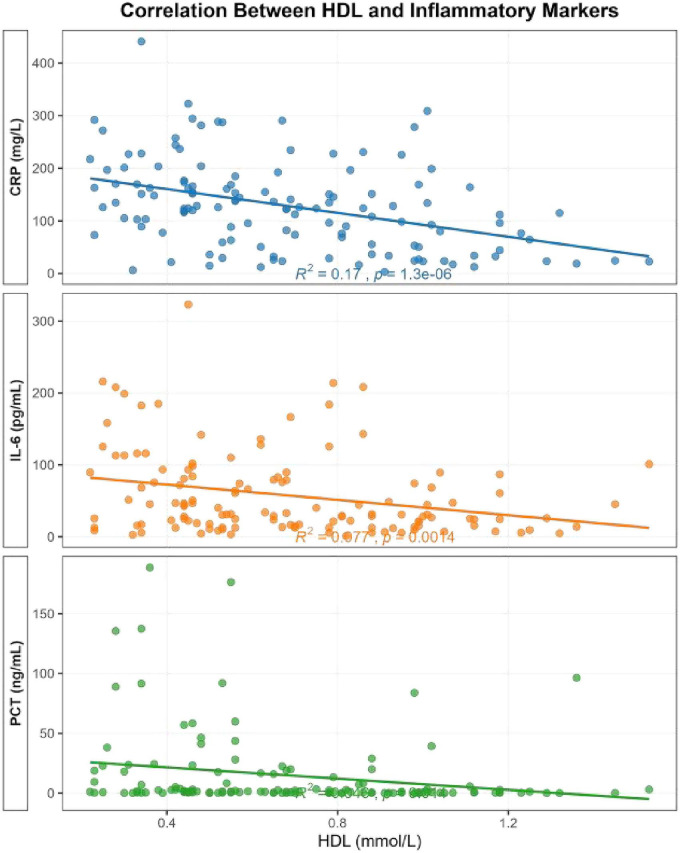
Correlation between HDL and inflammatory markers (CRP, IL-6, PCT).

In univariable analyses, HDL was negatively associated with CRP, IL-6, and PCT. In multivariable analyses, lower HDL remained independently associated with higher CRP and higher IL-6, whereas the association with PCT was not retained in the final multivariable model (Figure 2 and Table 2). Each dot in Figure 2 represents one patient in the primary cohort.

**TABLE 2:** Univariable and multivariable regression analyses.

Factor	β -coefficient	95% CI	*p*-value
Abscess size	0.112	(0.06, 0.16)	< 0.01
HDL	–12.4	(–16.29, –8.56)	< 0.01
Sepsis	3.08	(0.5, 5.66)	0.018
Pleurisy	4.98	(0.86, 9.1)	0.02
HIV	8.41	(0.77, 16.05)	0.03
**Variable**	**β -coefficient**	**95% CI**	***p*-value**
HDL	–16.20820335	(–20.17, –12.25)	4.29945E-13
Abscess size	0.164126427	(0.11,0.22)	2.24309E-07
LDL	–3.443738891	(–5, –1.89)	2.7708E-05
ALB	–0.438396341	(–0.66, –0.22)	0.000174366
IL-6	0.03909474	(0.01, 0.06)	0.002412984
Sepsis	4.925210084	(1.64, 8.21)	0.000125405
CRP	0.034189043	(0.02, 0.05)	0.004975514
Total cholesterol	–1.795456266	(–3.15, –0.44)	0.010387306
Neutrophils	0.284469339	(0.06, 0.51)	0.013218471
Pleuritis	6.461038961	(1.14, 11.79)	0.01878712
WBC	0.261432122	(0.04, 0.48)	0.023064603
FS	0.335478471	(0.04, 0.63)	0.025264719
Drainage	3.287878788	(0.04, 6.54)	0.049540094

**FIGURE 3:**
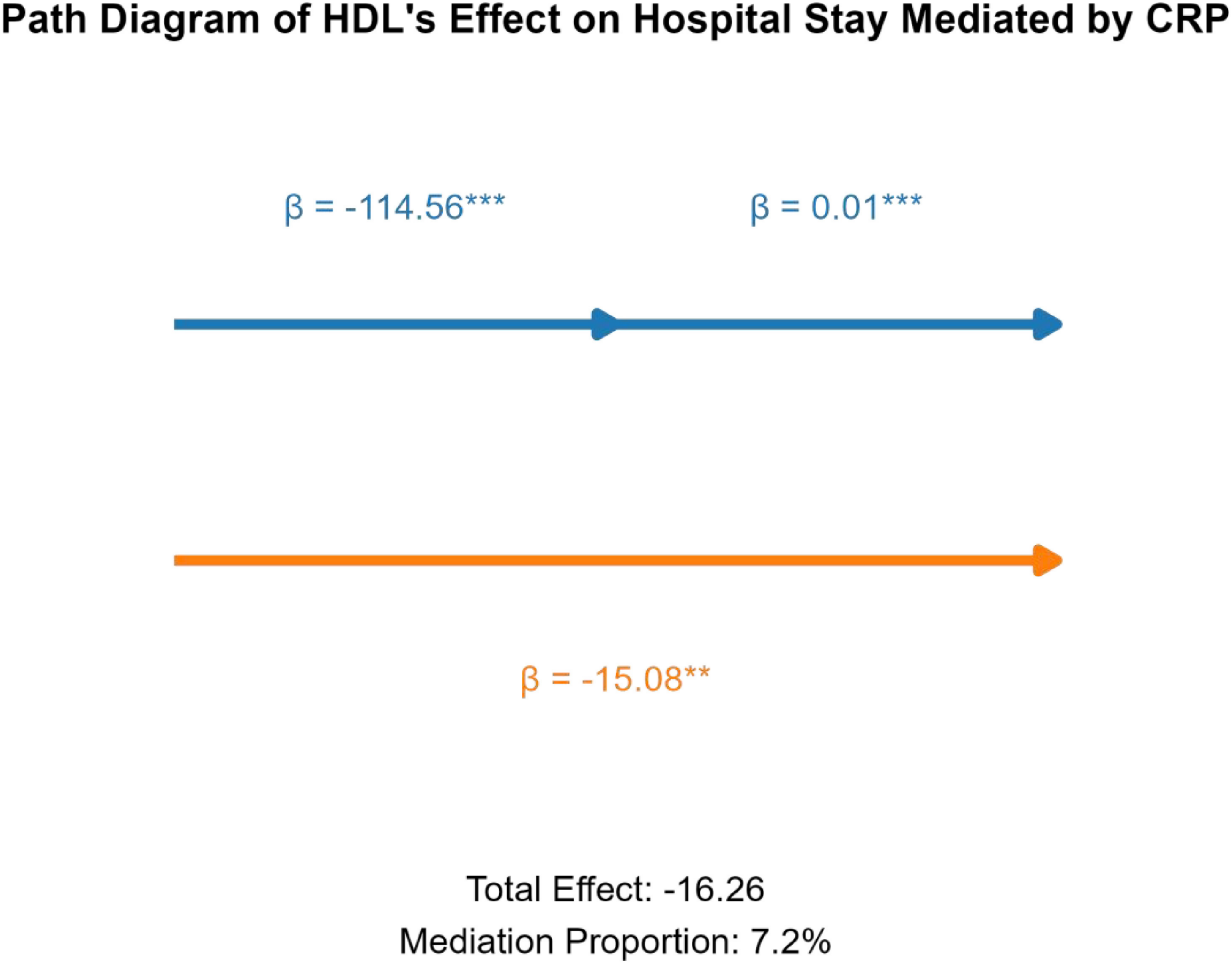
Mediation model: HDL → CRP → LOS.

Mediation analysis showed that CRP mediated 7.2% of the association between HDL and hospitalization duration. The total effect of HDL on hospitalization duration was −16.26 (Figure 3).

### Primary cohort predictive models

**FIGURE 4:**
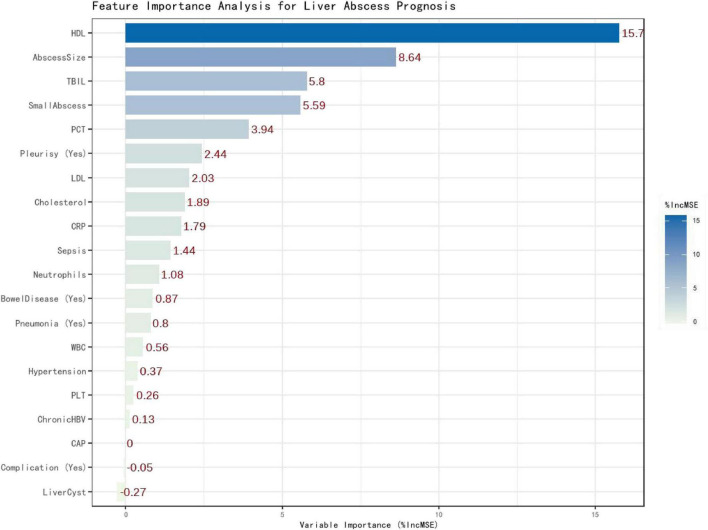
Variable importance in the random forest model.

**FIGURE 5:**
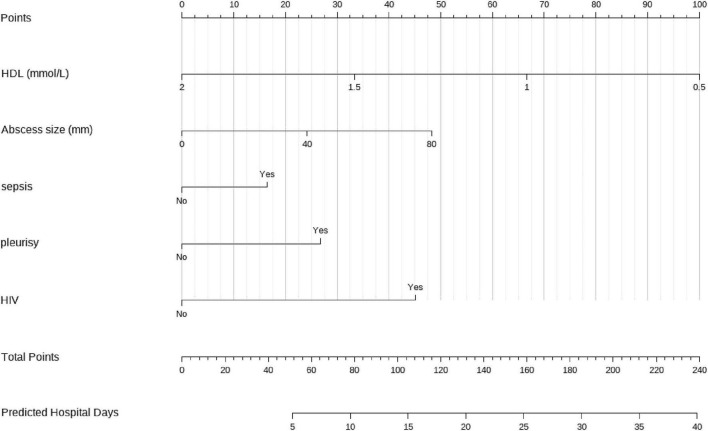
Nomogram for predicting hospitalization duration. Sepsis was defined as present at admission in the prospective cohort and by ICD-based coding in the MIMIC-IV cohort.

Random forest analysis ranked HDL as the variable with the highest importance for predicting hospitalization duration (%IncMSE = 15.75), followed by abscess size and total bilirubin (Figure 4). Based on the key predictors identified by Random Forest analysis, a multivariable linear regression model incorporating HDL, abscess size, and sepsis was developed and presented as a nomogram (Figure 5). The underlying regression model showed an R^2^ of 0.659 and an RMSE of 6.43 days (Table 3).

**TABLE 3:** Model performance comparison (RF, Nomogram).

Model	RMSE(95% CI)	*R*^2^/ρ (95% CI)
RF	6.8(6.20–7.40)	0.5 (0.450–0.572)
Nomogram	6.4(5.82–7.04)	0.6(0.601–0.717)

**FIGURE 6:**
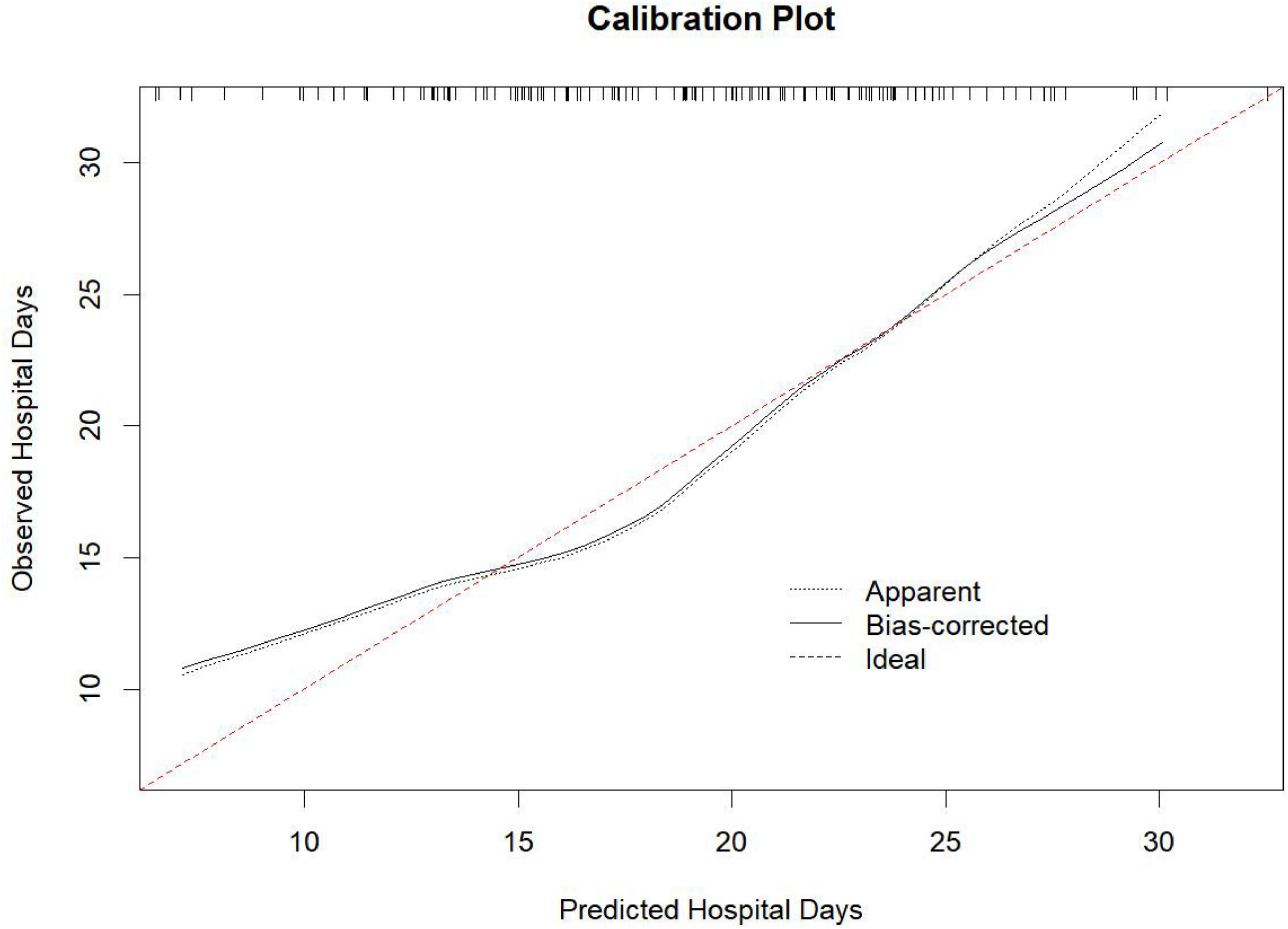
Calibration plot of the Nomogram model for predicting hospitalization duration calibration plot of the Nomogram model for predicting hospitalization duration. The x-axis represents the predicted hospitalization days by the Nomogram model. The y-axis represents the observed hospitalization days. Three curves are shown: Apparent curve: Model performance on the training data without bias correction (dotted line). Bias-corrected curve: Model performance after bootstrap bias correction (*B* = 1000; solid line). Ideal curve: Perfect agreement between predicted and observed values (dashed red line). The calibration intercept was −0.0 (95% CI: −3.6 to 3.6), and the calibration slope was 1.00 (95% CI: 0.82–1.18), indicating excellent model calibration.

Bootstrap validation (*B* = 1000) showed that the bias-corrected calibration curve aligned closely with the ideal line (Figure 6). The calibration intercept was −0.0 (95% CI: −3.6 to 3.6), and the calibration slope was 1.00 (95% CI: 0.82–1.18).

### MIMIC-IV cohort validation

In the MIMIC-IV validation cohort (*n* = 38), baseline demographic characteristics, including age, sex, and hospital LOS, are summarized in Supplementary Table 1. In multivariable linear regression analysis, lower HDL was independently associated with longer hospitalization duration (β = −0.98, 95% CI: −1.77 to −0.18, p = 0.017), while sepsis was positively associated with longer hospitalization duration (β = 26.34, 95% CI: 11.08–41.59, *p* < 0.001). Glucose, CRP, and diabetes were not significantly associated with hospitalization duration in the multivariable model (Table 4).

**TABLE 4:** Multivariable linear regression of factors associated with hospital length of stay in the MIMIC-IV validation cohort.

Factor	β -coefficient	95% CI	*p*-value
HDL (mg/dL)	−0.98	(-1.77, −0.18)	0.017
Glucose (mg/dL)	0.03	(–0.14, 0.21)	0.688
CRP (mg/dL)	0.14	(– 0.05, 0.32)	0.135
Sepsis (yes vs. no)	26.34	(11.08, 41.59)	< 0.001
Diabetes (yes vs. no)	−1.96	(-19.34, 15.42)	0.820

**FIGURE 7:**
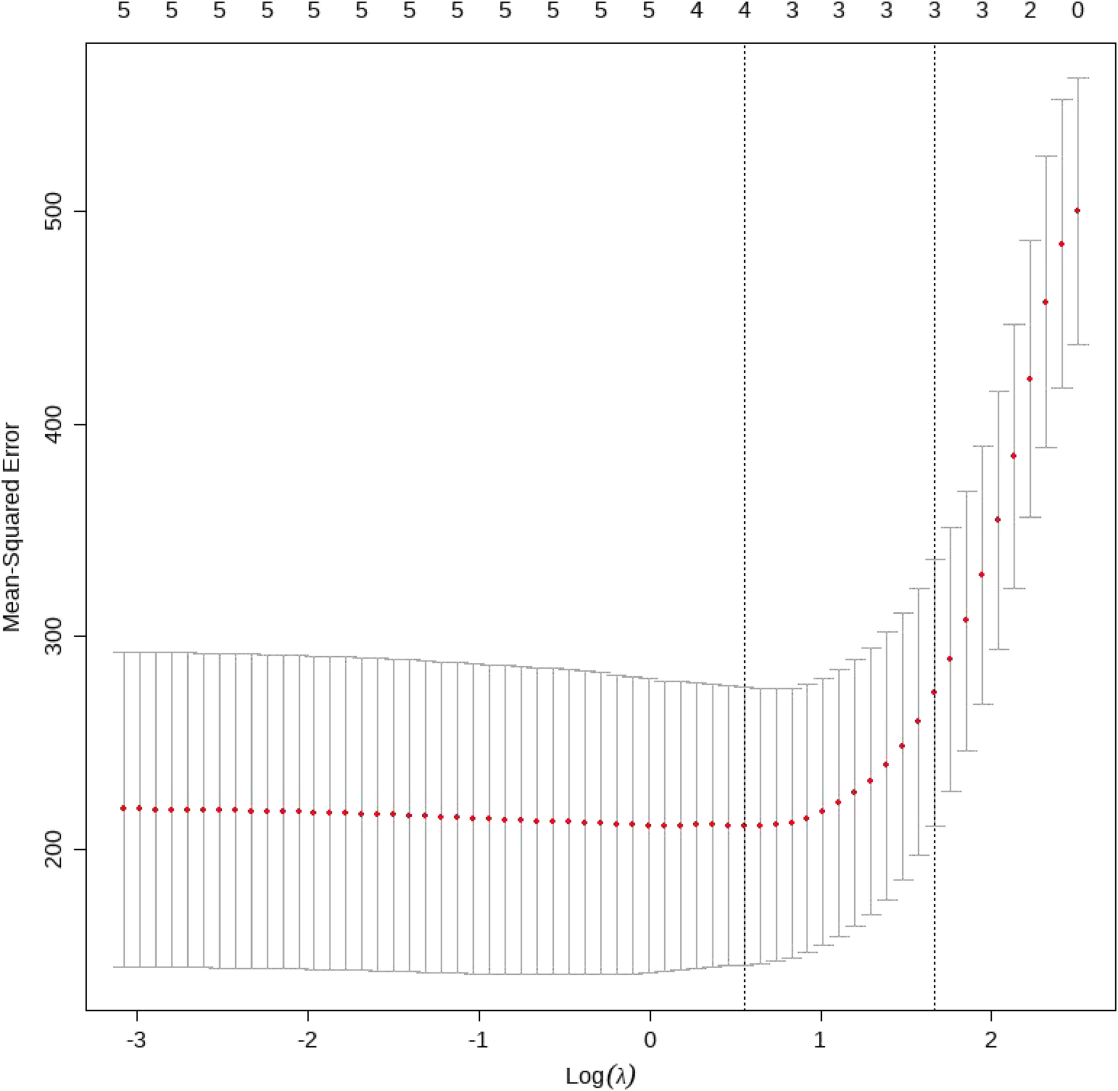
Cross-validation curve for LASSO regression in the MIMIC-IV cohort. X-axis: log(λ), the logarithm of regularization parameter. Y-axis: Mean Squared Error (MSE), quantifying model prediction error. Dashed vertical lines: Left line (lambda.min): Optimal λ value minimizing MSE. Right line (lambda.1se): Largest λ within one standard error of the minimum MSE, favoring simpler models. Red dots: Mean MSE across 5-fold cross-validation; error bars represent standard deviations. The corresponding coefficients are summarized in Table 5.

LASSO regression with 5-fold cross-validation identified HDL, CRP, and sepsis as variables with non-zero coefficients at λmin (Figure 7 and Table 5). The corresponding coefficients were HDL, β = −0.44 (95% CI: −0.62 to −0.26), CRP, β = 0.13 (95% CI: 0.05–0.21), and sepsis, β = 22.72 (95% CI: 18.35–27.09), all with *p* < 0.01.

**TABLE 5:** Variables retained in LASSO regression (λ = lambda.min).

Factor	β -coefficient
HDL	–0.44
CRP	0.13
Sepsis	22.72

Based on LASSO regression (Figure 7), HDL, CRP, and Sepsis were retained as key predictors of hospital length of stay.

### NHANES cohort analysis

In the NHANES cohort, baseline demographic characteristics are summarized in Supplementary Table 2. The combination of low HDL ( < 1.03 mmol/L or 40 mg/dL) and high CRP (>1.0 mg/dL) was associated with an increased risk of prolonged hospitalization (Table 6). Age and male sex were also associated with hospitalization risk. Restricted cubic spline analysis showed a dose-dependent decrease in hospitalization risk with higher HDL levels, particularly within the range of 0.5–1.5 mmol/L (Supplementary Figure 1).

**TABLE 6:** Logistic regression of factors associated with hospitalization (NHANES cohort).

Variable	95% CI	*p*-value
High-risk (HDL < 1.03 and CRP > 1.0)	1.22–1.77	< 0.001
Age	1.03–1.03	< 0.001
Sex	1.08–1.42	0.0018

## Discussion

In this multi-cohort study, lower admission HDL-C was consistently associated with longer hospitalization in patients with pyogenic liver abscess (PLA). This association remained evident in the primary cohort and was directionally supported by external analyses, suggesting that HDL-C may serve as a clinically accessible biomarker for early risk stratification. Because prolonged hospitalization in PLA is common and often difficult to anticipate at admission (3, 5, 7), these findings may be relevant for identifying patients who are likely to require closer monitoring and more complex inpatient management.

Our findings are also consistent with evidence from other infectious diseases (25) in which lower HDL levels have been associated with greater disease severity or worse outcomes (15, 16, 19). Prior reviews have highlighted the broader relevance of HDL in infectious diseases and sepsis (16–18), and recent evidence has further suggested that reduced circulating HDL-C is associated with poorer outcomes in sepsis and critical illness. Together, these observations support the interpretation of HDL as a host-response biomarker beyond PLA (26).

Among the clinical covariates, sepsis and pleural complications were associated with prolonged hospitalization (5–7, 27). These complications likely identify a subgroup of patients with greater systemic inflammatory burden, more severe physiologic derangement, and a more complicated disease course. Patients with sepsis may require broader antimicrobial treatment, closer monitoring, and longer time to clinical stabilization, while pleural complications may reflect more extensive regional inflammatory extension beyond the liver. These findings suggest that prolonged hospitalization in PLA is influenced not only by abscess burden itself, but also by the extent of systemic and extrahepatic complications.

The inverse associations between HDL and CRP, IL-6, and PCT support a relationship between HDL and inflammatory activity in PLA (16–18). In addition, CRP partially mediated the association between HDL and hospitalization duration (17, 28, 29), accounting for 7.2% of the observed effect. This suggests that inflammatory pathways contribute to the HDL–LOS relationship, although they do not fully explain it.

The external analyses further support the robustness of the main findings. In the MIMIC-IV validation cohort, lower HDL remained independently associated with longer hospitalization duration, whereas sepsis was also significantly associated with prolonged stay. In contrast, glucose, CRP, and diabetes were not independently associated with hospitalization duration in the final multivariable model. In the NHANES cohort, the combination of low HDL and high CRP was associated with an increased risk of prolonged hospitalization, suggesting that the prognostic relevance of HDL may extend beyond PLA to broader inflammatory contexts (18, 19, 30). Because data on diabetes subtype and long-term glycemic control were not consistently available, dysglycemia-related findings should be interpreted cautiously.

## Study limitations and future research

This study has several limitations. First, the MIMIC-IV validation cohort was relatively small, which may limit the precision of the external estimates. Second, although microbiological results were incorporated when available, not all patients had complete culture data. Third, the observational design precludes causal inference. Finally, detailed information on diabetes subtype and long-term glycemic control was not consistently available, limiting more detailed interpretation of glucose-related findings. Further prospective studies are needed to validate the implementation of HDL-C–based risk stratification in routine care and to clarify the biological pathways linking HDL to recovery in infectious disease.

## Conclusion

Lower admission HDL-C was associated with prolonged hospitalization in patients with pyogenic liver abscess. Admission HDL-C, particularly when interpreted together with CRP, may help identify patients at increased risk of longer hospital stay early in the admission course. This simple approach may support risk stratification, closer monitoring, and more proactive inpatient planning. Further prospective studies are needed to validate its implementation in routine clinical practice.

## Data Availability

The datasets presented in this study can be found in online repositories. The names of the repository/repositories and accession number(s) can be found in the article/Supplementary material.
