# Supplementary figures and images for: Optimizing healthcare resources in pyogenic liver abscess: a dual-threshold HDL-CRP model for predicting hospitalization duration across multi-cohorts
Source: Front Med (Lausanne). 2026 Apr 30;13:1708360. doi: 10.3389/fmed.2026.1708360 (PMC13171349; doi:10.3389/fmed.2026.1708360)

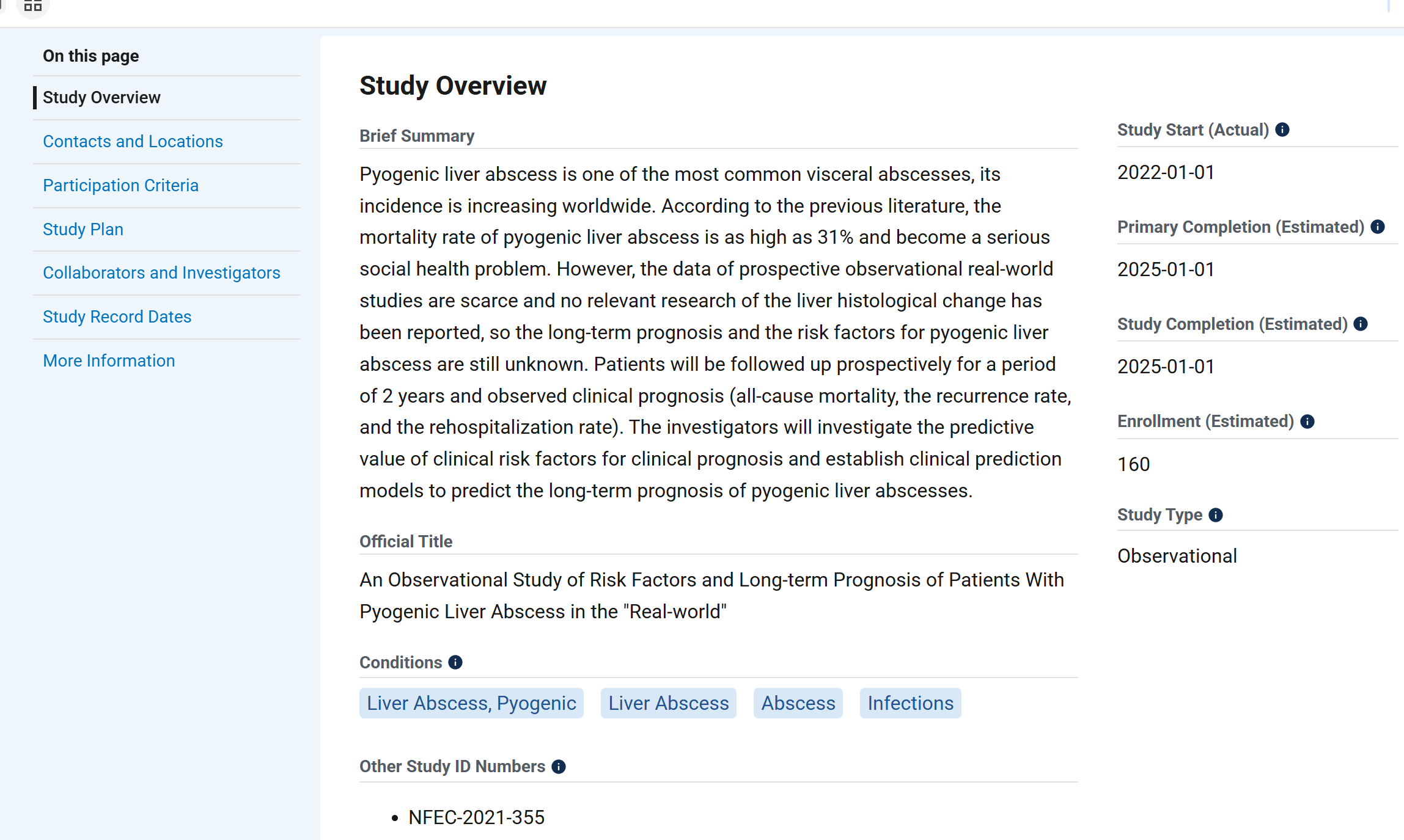

Supplement: Supplementary file 1 [file Data_Sheet_1.zip › “Supplementary File/clinicaltrials.png]
