# Supplementary material for: Optimizing healthcare resources in pyogenic liver abscess: a dual-threshold HDL-CRP model for predicting hospitalization duration across multi-cohorts
Source: Front Med (Lausanne). 2026 Apr 30;13:1708360. doi: 10.3389/fmed.2026.1708360 (PMC13171349; doi:10.3389/fmed.2026.1708360)
Supplement: Supplementary file 2 [file Data_Sheet_2.DOCX]

| Variable | Value |
| --- | --- |
| n | 38 |
| Age, years | 64.2 ± 13.8 |
| Male, n (%) | 20(52%) |
| Female, n (%) | 18(48%) |
| Hospital LOS, days | 33.7 ± 23.7 |

**Supplementary Table S1.**Baseline characteristics of the MIMIC-IV validation cohort

| Variable | Value |
| --- | --- |
| n | 9693 |
| Age, years | 49.6 ± 18.6 |
| Male, n (%) | 4718 (48.7%) |
| Female, n (%) | 4975 (51.3%) |
| HDL-C, mg/dL | 53.5 ± 15.9 |
| CRP | 4.16 ± 8.73 |
| Hospital LOS, days | 1.9 ± 0.3 |

**Supplementary Table S2.**Baseline characteristics of the NHANES 2017–2020 adult cohort

**
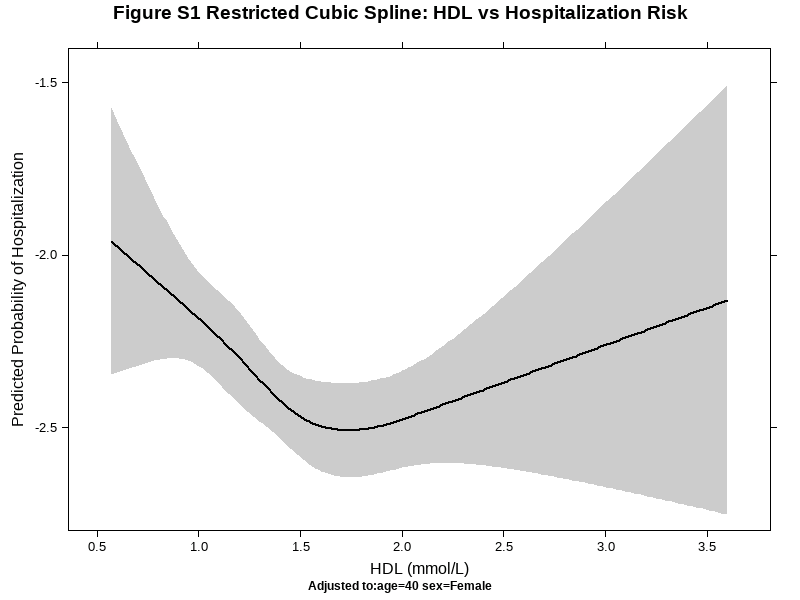
**

**Fig. S1.** Restricted cubic spline analysis of HDL and hospitalization risk (NHANES cohort).

Adjusted for age and sex. A restricted cubic spline model with four knots was used to explore the nonlinear association between HDL and the probability of hospitalization. Solid line represents estimated odds; dashed lines represent 95% confidence intervals.

**
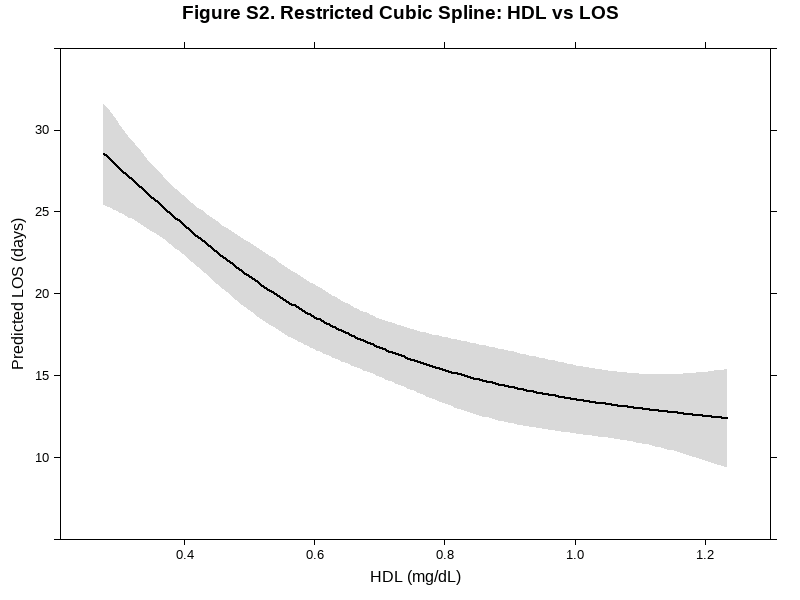
**

**Fig. S2.** Restricted cubic spline analysis of the association between HDL and hospital length of stay (mimic cohort).

A restricted cubic spline (RCS) model with four knots was used to assess the potential nonlinear association between baseline high-density lipoprotein (HDL) levels and predicted hospital length of stay (LOS). The solid line represents the estimated LOS, and the dashed lines represent the 95% confidence intervals.


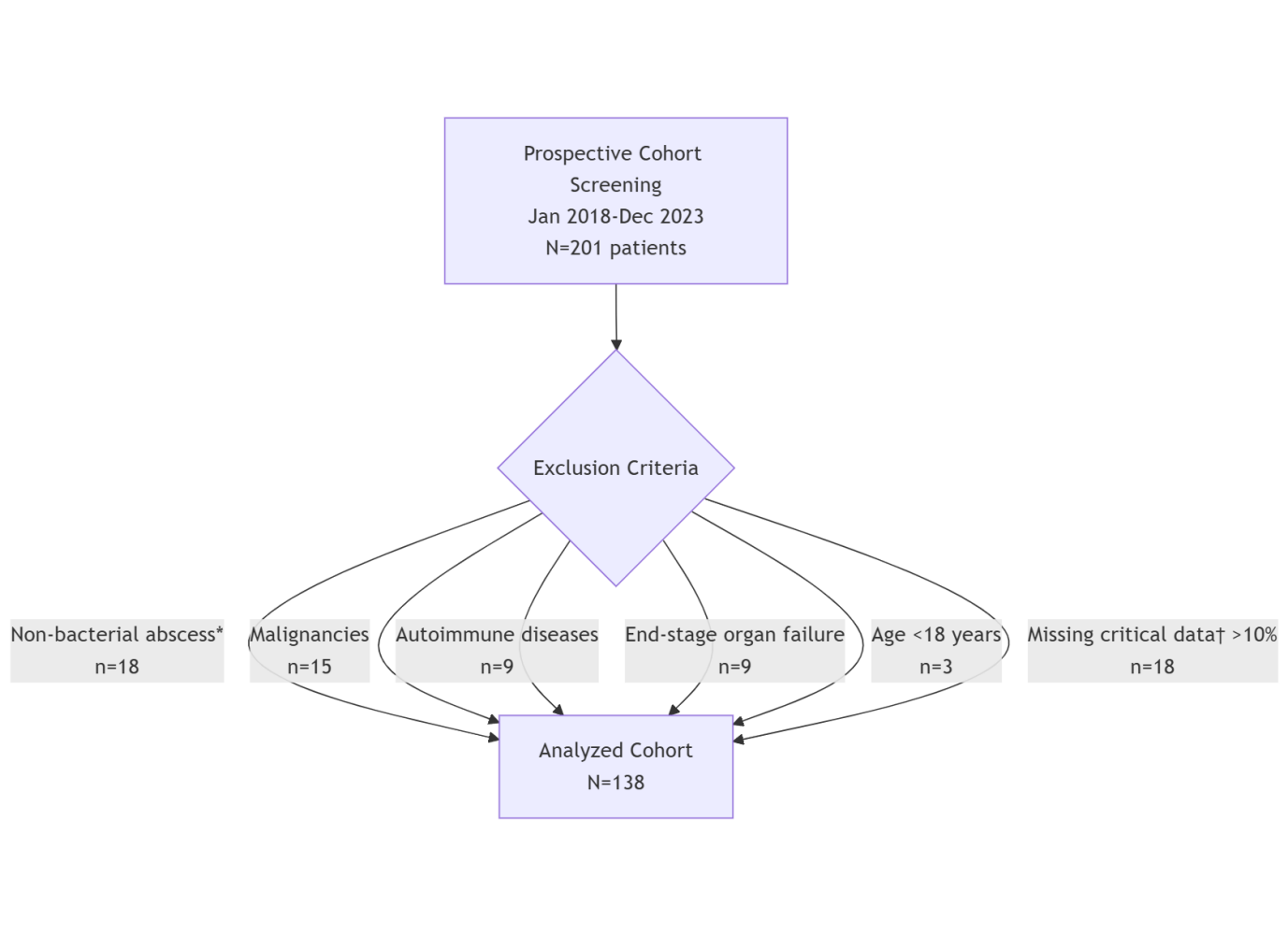


**Fig. S3.**ProspectiveCohortFlowchart

**
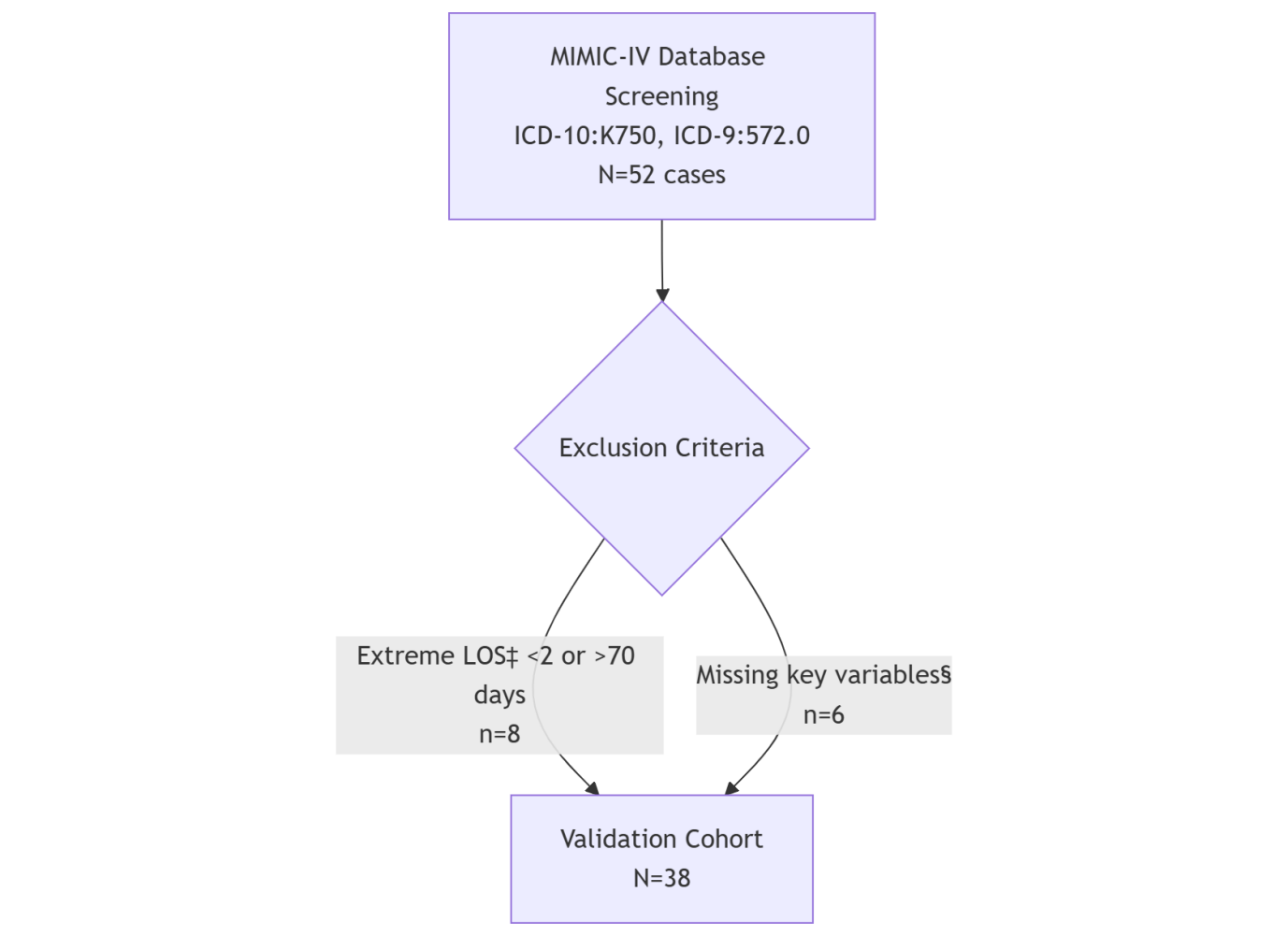
**

**Fig. S4.**MIMIC_IV_Flowchart

**
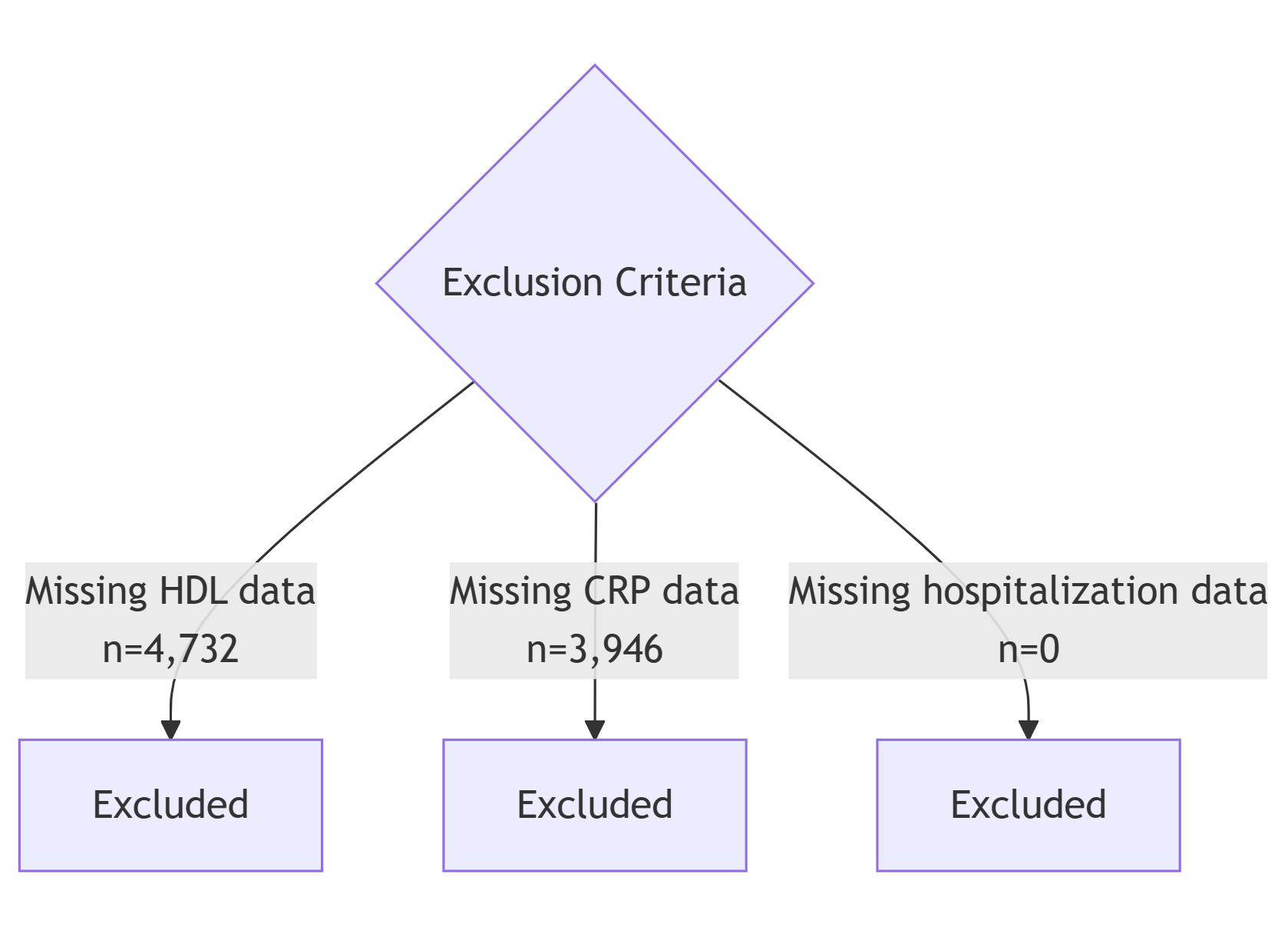
**

**Fig. S5.**NHANES_Exclusions
